# Treatment and Prognosis of Radiation-Associated Breast Angiosarcoma in a Nationwide Population

**DOI:** 10.1245/s10434-019-08085-1

**Published:** 2019-11-26

**Authors:** Samuli H. Salminen, Tom Wiklund, Mika M. Sampo, Maija Tarkkanen, Lea Pulliainen, Tom O. Böhling, Erkki Tukiainen, Katja Hukkinen, Carl P. Blomqvist

**Affiliations:** 1 Comprehensive Cancer Center, Helsinki University Hospital, Helsinki, Finland; 2 University of Helsinki, Helsinki, Finland; 3 Docrates Cancer Center, Helsinki, Finland; 4 Department of Pathology, University of Helsinki and HUSLAB, Helsinki University Hospital, Helsinki, Finland; 5 Department of Plastic Surgery, Helsinki University Hospital, Helsinki, Finland; 6 Department of Pathology, University of Helsinki, Helsinki, Finland; 7 Department of Radiology, Helsinki University Hospital, Helsinki, Finland

## Abstract

**Background:**

Radiation-associated angiosarcoma of the breast (RAASB) is an aggressive malignancy that is increasing in incidence. Only a few previous population-based studies have reported the results of RAASB treatment.

**Methods:**

A search for RAASB patients was carried out in the Finnish Cancer Registry, and treatment data were collected to identify prognostic factors for survival.

**Results:**

Overall, 50 RAASB patients were identified. The median follow-up time was 5.4 years (range 0.4–15.6), and the 5-year overall survival rate was 69%. Forty-seven (94%) patients were operated on with curative intent. Among these patients, the 5-year local recurrence-free survival, distant recurrence-free survival, and overall survival rates were 62%, 75%, and 74%, respectively. A larger planned surgical margin was associated with improved survival.

**Conclusions:**

We found that the majority of RAASB patients were eligible for radical surgical management in this population-based analysis. With radical surgery, the prognosis is relatively good.

Globally, breast cancer (BC) is the most commonly diagnosed cancer among women.^1^ Breast-conserving surgery combined with radiotherapy (RT) is the preferred local treatment of early-stage BC.^2^^,^^3^

Radiation-associated angiosarcoma of the breast (RAASB) is an uncommon severe complication of breast RT.^4^ In recent years, angiosarcoma has been the most common subtype of radiation-associated sarcoma of the breast.^5^ A newly published population-based study estimated the risk of RAASB to be approximately 0.1%.^6^ It has been postulated that the shift from radical mastectomies to breast-conserving surgery has resulted in secondary angiosarcomas arising in the breast.^7^

The main treatment of RAASB is surgical excision if diagnosed in a localized stage;^8^–^14^ however, different surgical approaches have previously been presented. It has been proposed that surgery should aim at resection of all previously irradiated skin^11^ with negative margins.^8^ However, on the other hand, one study concluded that breast-conserving surgery was non-inferior compared with mastectomy.^10^ In a previous study from our center, RAASB resection was aimed at a clinical side margin of more than 3 cm; at last follow-up, six of nine patients were disease-free.^15^ The benefit of adjuvant chemotherapy remains undetermined.^14^ In previous studies of RAASB, the 5-year overall survival (OS) rate has varied considerably, from 10% to 75%, respectively, likely due to the substantial heterogeneity in RAASB treatment and study populations.^6^^,^^8^^,^^12^^,^^16^^,^^17^ Most previously published research on RAASB treatment, except a recent population-based study,^6^ has mainly been performed in single tertiary cancer centers, which might have affected the results because of patient selection.

We set out to study the treatment and prognosis of RAASB in a Finnish nationwide population; RAASB patients were identified from the Finnish Cancer Registry (FCR). Treatment of localized RAASB was assessed and patients were followed for local or distant recurrences. Prognostic factors for local and distant recurrences, as well as OS, were analyzed.

## Methods

The FCR was searched for patients with previously diagnosed invasive BC (International Classification of Diseases for Oncology, Third Edition [ICD-O-3]^18^), and a subsequent angiosarcoma, during the period 1989–2014 (ICD-O-3^18^ morphology 9120, behavior 3), in, or close to, the breast or chest wall. The FCR was founded in 1952 and encompasses the entire population of Finland (currently approximately 5.6 million inhabitants). A regulation was administered by the National Board of Health in 1961 making the reporting of cancers to the FCR obligatory. BC and RAASB treatment data were gathered from treating hospitals. RAASB histopathology for the majority of cases was confirmed in a previous study.^5^

### Radiation-Associated Angiosarcoma of the Breast (RAASB) Treatment and Follow-Up Assessment

Patient records and pathology reports were assessed to document the planned surgical side margins according to the surgery report (cm), pathological surgical margins (cm), removed tissues (skin, subcutaneous tissue, mammary gland, pectoral fascia, pectoral muscle), and possible surgical reconstruction. The pathological surgical margin was defined as the measurement stated in the original pathology report. Size (defined as the longest diameter of macroscopic tumor or skin lesion) and grade (according to the French system^19^) were documented. Preoperative and postoperative adjuvant chemotherapy and RT were registered. Patients were followed up for local recurrences and distant metastases, and possible deaths and causes of death were registered.

### Statistical Analyses

Primary endpoints of the study were local recurrence-free survival (LRFS), distant recurrence-free survival (DRFS) and OS. LRFS was defined as the time from RAASB diagnosis to the first local recurrence; DRFS was defined as the time to first distant recurrence; and OS was defined as the time to death from any cause, with censoring at last follow-up. LRFS, DRFS, and OS were calculated using the Kaplan–Meier method, while prognostic factors for survival were analyzed using the Cox univariate model. IBM SPSS Statistics for Windows version 25 (IBM Corporation, Armonk, NY, USA) was used for all analyses.

### Ethics Approval and Consent to Participate

This study was approved by the Joint Ethics Committee of Helsinki University Hospital and the Ministry of Health and Social Affairs. All procedures performed in studies involving human participants were in accordance with the ethical standards of the institutional research committee and with the 1964 Helsinki declaration and its later amendments or comparable ethical standards. Due to the retrospective nature of this study, the requirement for obtaining informed consent was waived with the permission of the Joint Ethics Committee of Helsinki University Hospital and the Ministry of Health and Social Affairs.

## Results

Our search of the FCR yielded a total of 50 patients with RAASB; all patients were Caucasian. BC characteristics and treatment data are shown in Table 1. All patients had received RT for BC, and the majority had received a postoperative RT dose of 50 Gy in 2 Gy fractions (*n* = 38).

**Table 1:** Breast cancer characteristics

	*n* = 50	%
Surgery type		
Resection	39	78
Mastectomy	11	22
AJCC stage (8th edition)		
IA	30	60
IIA	9	18
IIB	8	16
IIIA	2	4
IIIB	1	2
Histology		
Ductal	35	70
Lobular	10	20
Mucinous	2	4
Tubular	2	4
Not assessed	1	2
Grade		
1	16	32
2	26	52
3	2	4
Not assessed	6	12
Estrogen receptor		
Positive	38	76
Negative	3	6
Not assessed	9	18
Progesterone receptor		
Positive	31	62
Negative	10	20
Not assessed	9	18
HER2 status		
Positive	1	2
Negative	20	40
Not assessed	29	58
Adjuvant endocrine therapy		
No	32	64
Yes	18	36
Adjuvant chemotherapy		
No	48	96
Yes	2	4

*AJCC* American Joint Committee on Cancer, *HER2* human epidermal growth factor receptor 2

### RAASB Characteristics

RAASB properties and treatment data are summarized in Tables 2 and 3. All patients had a localized disease at presentation, without distant metastases. Forty-seven (94%) patients were operated on with curative intent, of whom 10 (21%) were re-operated because of insufficient surgical margins (five insufficient margins not specified in the pathology report, three insufficient side and deep margins, one insufficient side margin, and one insufficient deep margin). The median pathological surgical margin was 2.0 cm (range 0–6.0). All patients had the skin of the breast and subcutaneous tissue excised. The surgical operation included the resection of the pectoral fascia and pectoral muscle in 70% and 32% of patients, respectively. Twenty-four (51%) patients had a surgical reconstruction. The tissue defect was most commonly reconstructed with a pedicled latissimus dorsi flap with (seven patients) or without (eight patients) skin grafts. Three patients with RAASB were not operated on, two because of the extent of disease and impaired health, and one because of a metastatic BC. These three patients all died after a median of 44 days (range 23–58).

**Table 2:** RAASB characteristics

	*n* = 50	%
Age at RAASB diagnosis, years [mean (SD)]	70.4 (SD 9.1)	
RAASB latency, years^a^ [median (range)]	7.7 (0.6–24.5)	
Size based on histopathologic report, cm [median (range)]	4.0 (0.5–16.0)	
Not assessed	16	32
Location		
Breast	40	80
Mastectomy scar	5	10
Upper trunk	5	10
Grade (according to the French system^19^)		
2	6	12
3	38	76
Not assessed	6	6

*RAASB* radiation-associated angiosarcoma of the breast, *SD* standard deviation

^a^RAASB latency was defined as the time from radiotherapy for breast cancer to RAASB diagnosis

**Table 3:** Radiation-associated angiosarcoma of the breast treatment characteristics

	*n* = 47	**%**
Planned surgical side margin, cm [median (range)]	4.0 (range 0.5–5.0)	
Not specified	16	
Pathological surgical margin, cm [median (range)]	2.0 (range 0.0–6.0)	
Not assessed	12	
Surgically removed tissues		
Skin of breast	47	100
Subcutaneous tissue	47	100
Mammary gland	37	79
Pectoral fascia	33	70
Pectoral muscle	15	32
Reconstruction		
No reconstruction	23	49
Latissimus dorsi flap	8	17
Latissimus dorsi flap with skin graft	7	15
Pedicled transverse rectus abdominis myocutaneous flap	2	4
Pedicled transverse rectus abdominis myocutaneous flap with skin graft	1	2
Pedicled thoracolateral flap	1	2
Only skin graft	5	11
Postoperative radiotherapy		
No	46	98
Yes	1	2
Postoperative chemotherapy		
No	42	89
Paclitaxel	3	6
Doxorubicin	1	2
Docetaxel and fluorouracil/epirubicin/cyclofosfamide^a^	1	2

^a^Erroneously diagnosed as breast cancer recurrence

Five patients received postoperative chemotherapy, of whom three received paclitaxel, one received doxorubicin, and one received a taxane- and anthracycline-based regimen after an erroneous diagnosis of BC recurrence. This patient also received postoperative RT. The most common indication for postoperative chemotherapy was a large tumor size.

**Fig. 1:**
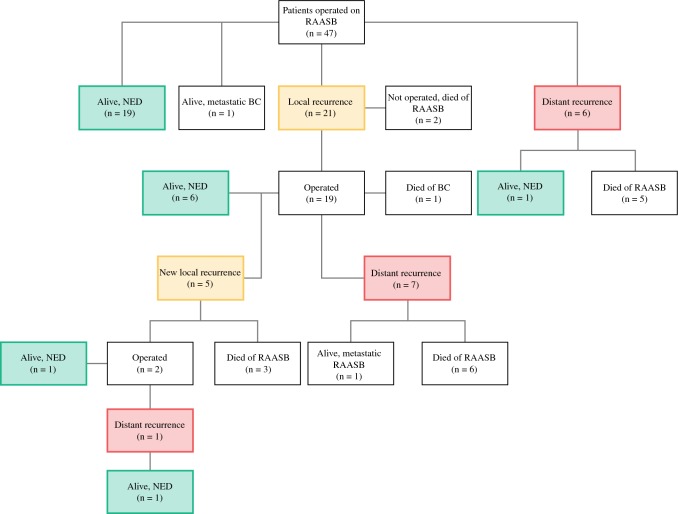
Treatment and outcome of curatively treated RAASB patients. *BC* breast cancer, *NED* no evidence of disease, *RAASB* radiation-associated angiosarcoma of the breast

The treatment and outcome of patients operated with curative intent (*n* = 47) are summarized in Fig. 1. The median follow-up time was 5.4 years (range 0.4–15.6). No patients were lost to follow-up, and 19 (40%) patients were alive without any RAASB recurrence at last follow-up. A local recurrence of RAASB was diagnosed in 21 of 47 (45%) patients after a median of 1.0 years (range 0.1–10.0); 19 (90%) of these patients were operated on with curative intent, 6 of whom remained free of local and distant recurrence at last follow-up. One additional patient has been free of local and distant recurrence following surgery for a second local recurrence. The median margin, at surgery, of local recurrences was 1.0 cm (range 0.05–3.0). At the last follow-up, 7 (33%) of the 21 patients with a local recurrence were alive without evidence of disease, 1 (5%) was alive with a metastatic RAASB treated with paclitaxel, 1 (5%) was alive with a metastatic RAASB, 11 (52%) had died of RAASB, and 1 (5%) had died of BC.

A distant metastasis of RAASB was diagnosed in 14 (30%) patients after a median of 1.1 years (range 0.4–10.5), 8 (57%) of whom had and 6 (43%) did not have a preceding or simultaneous local recurrence. At last follow-up, 11 of these 14 (79%) patients had died of metastatic RAASB and 3 patients were alive with a metastatic RAASB, 2 with no evidence of disease (4.4 and 5.1 years after the diagnosis of a metastatic RAASB to the lymph nodes and treatment with paclitaxel) and 1 with ongoing treatments for metastatic disease. The most common metastatic sites were the lungs (*n* = 8), liver (*n* = 4), and lymph nodes (*n* = 4). Four patients had multiple metastatic sites.

**Fig. 2:**
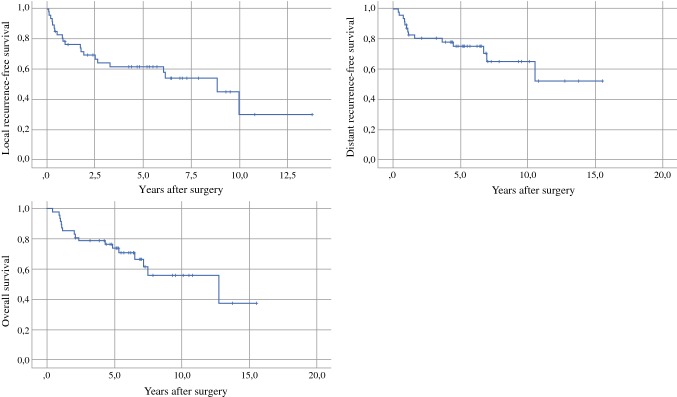
Kaplan–Meier curves of local recurrence-free survival, distant recurrence-free survival, and overall survival in patients operated on with curative intent (*n* = 47)

For operated patients (*n* = 47), the 5-year LRFS, DRFS, and OS rates were 62%, 75%, and 74%, respectively (Fig. 2). For all patients (*n* = 50), the 5- and 10-year OS rates were 69% and 52%, respectively. Prognostic factors for survival in operated patients, determined using a Cox univariate analysis, are presented in Table 4. Only the planned surgical side margin (hazard ratio [HR] 0.419 per cm, 95% confidence interval [CI] 0.236–0.744; *p* = 0.003) was associated with the risk of death. There was a weak positive correlation between the planned surgical margin and the margin assessed by the pathologist (*R*_s_ = 0.391; *p* = 0.059). None of the studied factors were significantly associated with local or distant recurrences, however there was a non-significant trend towards improved local control in patients operated with reconstructive surgery (HR 0.454, 95% CI 0.182–1.133; *p* = 0.091).

**Table 4:** Cox univariate analysis of hazard ratios for local recurrence-free survival, distant recurrence-free survival, and overall survival in radiation-associated angiosarcoma of the breast patients operated on with curative intent (*n* = 47)

	LRFS			DRFS			OS		
	*p*	HR	95% CI	*p*	HR	95% CI	*p*	HR	95% CI
Breast cancer surgery type (mastectomy vs. resection)	0.265	0.561	0.203–1.551	0.829	1.181	0.261–5.337	0.411	0.621	0.200–1.931
Age at RAASB diagnosis^a^	0.126	1.046	0.987–1.109	0.187	1.048	0.978–1.123	0.389	1.027	0.967–1.091
Year of RAASB diagnosis^a^	0.337	0.958	0.877–1.046	0.169	1.097	0.961–1.253	0.873	0.992	0.899–1.094
RAASB latency (years)^a^	0.691	1.022	0.918–1.139	0.087	1.120	0.984–1.275	0.160	1.093	0.965–1.238
Tumor size according to the pathologist’s report (cm)^a^	0.814	1.014	0.906–1.134	0.253	1.075	0.950–1.216	0.413	1.054	0.929–1.196
Grade (2 vs. 3)	0.546	0.676	0.190–2.410	0.379	25.914	0.018–36,636.3	0.414	25.763	0.011–62,885
Planned surgical side margin (cm)^a^	0.184	0.685	0.392–1.197	0.470	0.796	0.429–1.477	0.003	0.419	0.236–0.744
Surgical margin according to the pathologist’s report (cm)^a^	0.558	0.906	0.650–1.262	0.177	0.714	0.438–1.165	0.090	0.677	0.431–1.063
Extent of surgery (only skin/subcutaneous tissue vs. removal of muscle/fascia)	0.260	0.591	0.237–1.475	0.993	1.005	0.318–3.172	0.283	0.581	0.215–1.568
Reconstructive surgery (no vs. yes)	0.091	0.454	0.182–1.133	0.293	0.555	0.185–1.663	0.266	0.567	0.209–1.540
Postoperative radiotherapy (no vs. yes)	0.686	0.048	0.000–120,815	0.125	5.103	0.638–40.833	0.119	5.232	0.654–41.850
Postoperative chemotherapy (no vs. yes)	0.993	1.007	0.230–4.407	0.521	1.652	0.357–7.653	0.235	2.170	0.604–7.805

*HR* hazard ratio, *CI* confidence interval, *LRFS* local recurrence-free survival, *DRFS* distant recurrence-free survival, *OS* overall survival, *RAASB* radiation-associated angiosarcoma of the breast

^a^Continuous variable

## Discussion

Breast-conserving surgery and RT constitute the preferred local treatment of early BC. RAASB is a serious but fortunately uncommon complication of RT. The current study assessed RAASB treatment and prognosis in a nationwide population. We found that a radical surgical operation with curative intent is possible in the majority of RAASB patients. The 5- and 10-year OS rates of 69% and 52%, respectively, compare favorably with previous studies, mostly institutional series, reporting 5-year OS rates of 10–75%,^8^^,^^10^^,^^12^^,^^20^ and a recent large (*n* = 209) nationwide study reporting 5- and 10-year OS rates of 41% and 25%, respectively.^6^ The large variation in survival is presumably derived from the diverse patient populations.

The primary treatment of localized RAASB is surgery. Awareness and early diagnosis of RAASB is crucial to enable surgical treatment with curative intent. In the present study, almost all patients (94%) underwent surgery for RAASB. The type of surgery, amount of removed tissues, and methods of surgical reconstruction varied considerably, reflecting the nationwide patient population and variable policies of treating hospitals. Previous research has emphasized the importance of extensive radical operations to improve survival.^11^^,^^13^ In a study of 38 RAASB patients, 36 (95%) patients were operated on with curative intent,^13^ of whom 23 had a radical excision with surgical reconstruction and 13 had a non-radical excision. The authors observed a greater number of local recurrences in patients with < 1 cm margins, and concluded that at their institution, RAASB management currently involves a radical excision of the irradiated breast area. In a retrospective analysis from a tertiary center, a simple mastectomy alone was performed in 4 of 13 surgically treated patients.^8^ In the current study, the practice of removing the irradiated breast area was not followed. Instead, the surgical treatment consisted of a radical excision of the lesion with a median planned surgical side margin of 4.0 cm. The removal of all the irradiated breast area may require extensive and mutilating surgical operations and surgical reconstruction. One study has suggested that radiation-associated sarcomas arise in or at the edge of the radiation field.^21^ This view was further supported by a recent study that proposed that RAASB “arise from a field change within the irradiated tissue”.^13^ In the current study, the tissue defect was most often covered with pedicled latissimus dorsi flaps; however, detecting an RAASB recurrence can be more difficult if a pedicled flap is used. On the other hand, skin grafts may allow for a more reliable follow-up of the affected area.

We found that a larger planned surgical side margin was prognostic for OS, and there was also a non-significant trend towards an improved OS for the margin measured in the pathologist’s report. The weaker association with the pathologist’s assessment of the margin may reflect the difficulty in determining the extent of RAASB due to the multifocal growth pattern of RAASB, as previously postulated.^11^ While surgical margins were not statistically significantly associated with local or distant recurrences, they showed weaker trends towards a better outcome, with wide CIs. Some previous studies have not found any association between the surgical margin and a local recurrence^9^^,^^14^ or OS.^20^^,^^22^ On the other hand, a study including both primary and secondary angiosarcomas of the breast reported an improved OS for patients with negative surgical margins.^23^ Moreover, one study defining radical margins as removal of all or nearly all previous irradiated breast skin reported an improved 5-year disease-specific survival of 86%, versus 46% for radically versus conservatively operated patients.^11^ It is important to note that most individual studies, including the present study, are small, making assessment of prognostic factors unreliable. Due to its retrospective nature, the current study is missing information regarding the planned surgical side margin and pathological surgical side margin in a number of patients. It is important to bear in mind the possible bias in these results. A meta-analysis of published data comprising 222 RAASB patients reported that tumor size (*p* < 0.0005) and combination treatment with surgery and irradiation (*p* = 0.01) improved local control significantly, while tumor size (*p* < 0.0005), and age (*p* = 0.048), were prognostic for OS.^24^ Surgical margins were not assessed in this meta-analysis.

A notable finding in the current study was the high risk of local RAASB recurrence (45%). Ninety percent of these patients underwent salvage surgery, and, at last follow-up, only 6 of 21 (29%) patients remain alive without further recurrences. Previous investigations of RAASB have reported local recurrence rates ranging from 30 to 92% of patients.^8^^,^^9^^,^^12^^–^14^,^^17^ Unfortunately, only a few of these studies provide further follow-up data after local recurrence. In a study of 79 RAASB patients, only 25% of patients with a local recurrence later succumbed to RAASB.^14^ Therefore, the authors suggested an intensive treatment of local recurrences to improve survival. In another study, a local recurrence was found in 19 of 31 (61%) patients,^22^ of whom 11 had a surgical excision of the recurrence, with a median survival of 34 months compared with 6 months in patients with no surgical intervention. Our results corroborate the previous findings and further support a radical surgical approach with curative intent in a local recurrence. In our institution, the follow-up plan also includes photographs of the affected area. The initial follow-up interval is short, with gradual prolongation if no recurrences occur.

In the current study, only one patient received postoperative RT and five patients received postoperative chemotherapy. In previous studies, the use of pre- or postoperative chemotherapy has ranged from 3% to 45% and no firm conclusions can be drawn regarding its beneficial impact.^11^^,^^12^^,^^14^^,^^17^^,^^20^^,^^22^ Administration of RT for RAASB is controversial due to the previously delivered RT for BC and the presumably RT-induced etiology of RAASB. In the literature, the application of pre- or postoperative RT has ranged substantially from 4 to 35%.^10^^,^^12^^,^^20^^,^^25^ Whether RT was administered pre- or postoperatively is often not declared. One study reported an improved disease-free survival at 2 years after adjuvant RT (explicit information on RT schedule was not disclosed), however both primary breast angiosarcoma and RAASB were included and the study population was small (*n* = 35).^25^ In another population-based study of exclusively RAASB, no survival advantage was reported for patients managed with both surgery and RT compared with surgery alone.^6^

The results of our report are subject to at least three limitations. First, this was a retrospective study and it is possible that reporting to the nationwide cancer registry has been incomplete. However, this is unlikely because the FCR has an estimated coverage of 96% in all solid tumors and 82.9% in tumors of bone and soft tissues.^26^ Second, treatment policies may have changed during the study period. However, the year of RAASB diagnosis did not affect prognosis. Third, although relatively large, our patient population is underpowered to detect prognostic factors with a small or moderate impact. The most important strengths originate from the population-based patient cohort, systematic confirmation of RAASB from hospital and pathology records, and complete follow-up of all patients. RAASB treatment in Finland is mainly concentrated in the five university hospitals; however, some patients are treated in local hospitals. It is therefore possible that RAASB treatment protocols may differ and this may influence the results observed in this research.

## Conclusions

The present nationwide analysis of RAASB indicates that a radical resection with wide margins was achievable in most cases. The primary surgical intervention should aim at the best possible local control because a significant proportion of patients experiencing a local recurrence further develop a metastatic disease. The 5-year OS of 74% in surgically operated patients is higher than in most previous series. Planned excision with wide surgical margins was associated with an improved prognosis. The expected growth in the number of BC survivors may possibly lead to a further increase in RAASB incidence. Awareness of RAASB as a late side effect of RT for BC is essential to facilitate an early diagnosis, enable a radical surgical treatment, and eventually improve the prognosis. Consolidating RAASB treatment to high-volume centers is advisable to ensure quality of care.

## Data Availability

The datasets of the current study can be made available from the corresponding author upon reasonable request.
